# Timing-Dependent Effects of Dexamethasone in a Mouse Model of Neonatal Hypoxic–Ischemic Brain Injury: A Transcriptomic Analysis of Synaptic Signaling and Calcium Homeostasis

**DOI:** 10.3390/ijms27114920

**Published:** 2026-05-29

**Authors:** Joohee Lim, Jungho Han, Jeung Eun Shin, Kwangsoo Jung, Il-Sun Kim, Younhee Ko, Kook In Park

**Affiliations:** 1 Department of Pediatrics, Yonsei University College of Medicine, Seoul 03722, Republic of Korea; imagine513@yuhs.ac (J.L.); feagd@yuhs.ac (J.H.); golden-week@yuhs.ac (J.E.S.); whitesnown7@yuhs.ac (I.-S.K.); 2 Research and Development Team, Radexel Inc., Seoul 04387, Republic of Korea; im.kwangsoo.jung@gmail.com; 3 Division of Biomedical Engineering, Hankuk University of Foreign Studies, Yongin 17035, Republic of Korea

**Keywords:** neonatal hypoxic–ischemic brain injury, dexamethasone, neuroprotection, transcriptomics, synaptic signaling, calcium homeostasis

## Abstract

The optimal timing and therapeutic role of dexamethasone for neuroprotection in neonatal hypoxic–ischemic (HI) brain injury remain unclear. We investigated whether dexamethasone-mediated neuroprotection is time-dependent and explored its underlying molecular mechanisms in a neonatal HI mouse model. The Rice–Vannucci model (unilateral carotid artery ligation followed by 8% O_2_ for 90 min) was constructed utilizing postnatal day 7 mice who received vehicle (*n* = 5), dexamethasone pre-treatment (0.5 mg/kg, 6 h before HI; *n* = 6), or dexamethasone post-treatment (0.5 mg/kg, 6 h after HI; *n* = 6). Brain injury severity was evaluated by two blinded investigators 72 h after HI, who measured the whitish discoloration in the ipsilateral hemisphere. Transcriptomic analysis was performed using five representative brain samples from each group. Dexamethasone pre-treatment significantly reduced the area of whitish discoloration compared with the vehicle (*p* < 0.001); dexamethasone post-treatment exerted no significant protective effect. Transcriptomic profiling identified 962 (407 upregulated and 555 downregulated) differentially expressed genes. Genes with upregulated expressions were enriched in pathways related to central nervous system development, synaptic signaling, and calcium homeostasis; those with downregulated expressions were associated with cellular metabolic processes. Protein–protein interaction network analysis identified *Dlg4*, *Calm1*, and *Grin1* as hub genes. qRT-PCR validation confirmed significant upregulation of *Grin1* and *Calm1*, whereas *Dlg4* showed a concordant but non-significant trend. These findings suggest that dexamethasone pre-treatment may be associated with time-dependent changes in synaptic- and calcium-related gene expression following neonatal HI injury, providing insight into the optimal therapeutic window for neonatal HI brain injury.

## 1. Introduction

Hypoxic–ischemic (HI) brain injury is a major cause of neonatal mortality and severe long-term neurological morbidity. HI brain injury occurs in 1–8 cases per 1000 births [1]. The pathogenesis of HI brain injury is complex and involves short-term neuronal damage that evolves into long-term chronic inflammation [2]. Neonatal HI brain injury induces cell death, which is exacerbated by the abnormal expression and activation of the ATP-dependent Na+/K+ pump, oxidative stress, and aberrant excitatory neurotransmission because of insufficient oxygen and blood flow. Furthermore, the loss of highly vulnerable axons, oligodendrocyte progenitors, and neurons disrupts the maturation of neural networks, with a destructive impact on the brain’s structure and connectivity [3,4,5].

Currently, therapeutic hypothermia is the only established treatment for moderate to severe HI brain injury; however, its neuroprotective effects are limited, particularly with respect to long-term motor, sensory, cognitive, and behavioral outcomes [6,7]. Therefore, there is a critical need for adjunctive therapies that can enhance the efficacy of therapeutic hypothermia. Various candidate agents, including xenon, erythropoietin, melatonin, azithromycin, caffeine, cannabinoids, stem cell therapy, and granulocyte colony-stimulating factor (G-CSF), have been investigated for their potential neuroprotective and anti-inflammatory effects. In addition, glucocorticoids such as dexamethasone have also been explored as potential therapeutic agents in experimental models [8].

Neuroinflammation is a key contributor to the pathophysiology of perinatal brain injury, and glucocorticoids have been implicated in modulating inflammatory responses following acute cerebral HI [9,10,11]. Glucocorticoids are steroid hormones that regulate metabolism and immune responses. Dexamethasone, a synthetic glucocorticoid with high affinity for glucocorticoid receptors, is widely used in neonates both antenatally and postnatally [12,13,14]. However, their effects in neonatal HI brain injury remain controversial. Although some studies have reported deleterious effects, including impaired white matter development because of reduced myelin thickness and axonal caliber, glucocorticoids have also been shown to exert neuroprotective effects depending on the timing, dose, and duration of exposure [15]. Notably, experimental studies indicate that dexamethasone pre-treatment reduces the severity of brain injury, including infarct volume, and improves functional outcomes, likely via modulation of inflammatory and apoptotic pathways [10,16,17,18]. Despite these findings, the precise molecular mechanisms underlying glucocorticoid-mediated neuroprotection remain incompletely understood, although pathways such as PI3K/Akt, VEGF, CXCR4, and ERK have been implicated [10,11,18,19]. In particular, the transcriptomic effects of dexamethasone in neonatal HI brain injury, especially in relation to the timing of administration, have not been fully elucidated.

Although animal models have provided important insights into neonatal HI brain injury and glucocorticoid-mediated effects, their translational applicability is limited by species-specific differences and variability in injury mechanisms [20]. Therefore, unbiased approaches such as transcriptomic analysis are needed to better characterize the underlying molecular mechanisms. Recent studies have demonstrated that targeting inflammation- and oxidative stress-related pathways may confer neuroprotection in neonatal HI brain injury. For instance, (+)-JQ1 suppresses cGAS/STING-mediated inflammation via SIRT3, whereas arctiin reduces oxidative stress and restores Neuregulin-1 signaling, thereby improving neurological outcomes [21,22]. These findings highlight the importance of pathway-level modulation and support the need for comprehensive transcriptomic approaches to identify key regulatory mechanisms.

In this study, we investigated the neuroprotective effects of dexamethasone in a neonatal HI brain injury model, with a particular focus on the timing of administration (pre-treatment versus post-treatment). Using high-throughput RNA sequencing, we analyzed differentially expressed genes (DEGs), their associated pathways, and protein–protein interaction (PPI) networks. Using this comparative approach, we aimed to determine whether the neuroprotective effects of dexamethasone are dependent on the timing of administration and to identify transcriptomic changes associated with its efficacy. Key findings were further evaluated using quantitative real-time polymerase chain reaction (qRT-PCR).

## 2. Results

### 2.1. Effects of Dexamethasone on Gross Brain Morphology After Hypoxic–Ischemic Injury

**Figure 1:**
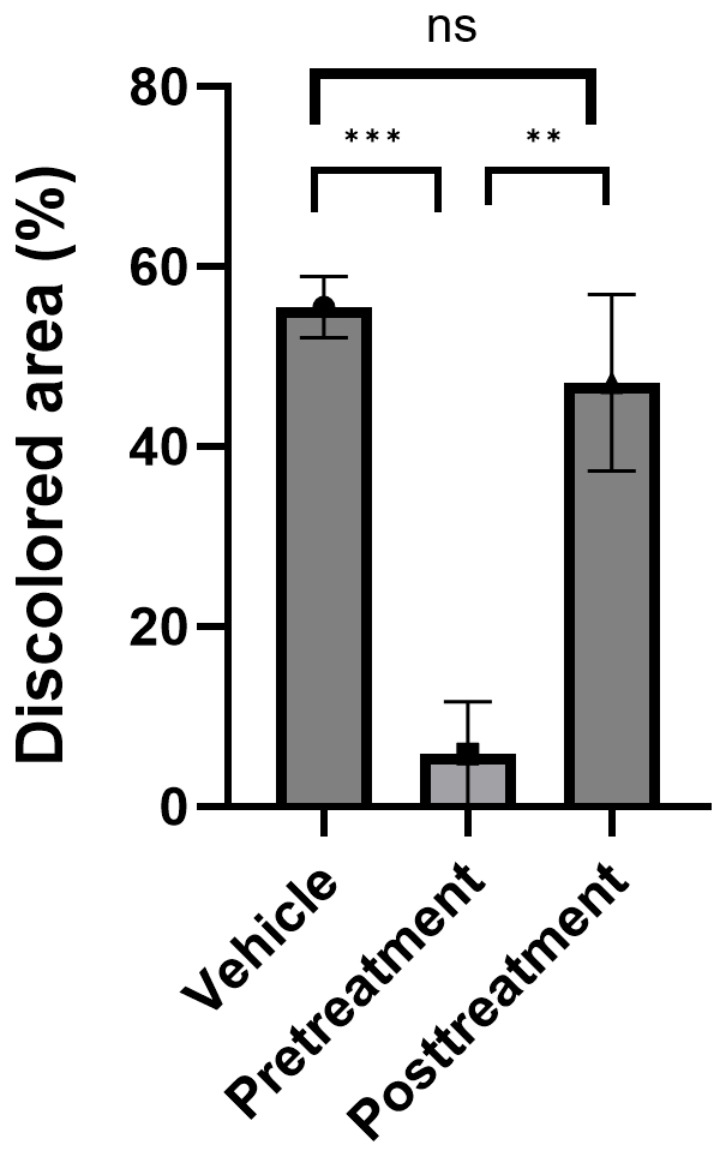
Effects of dexamethasone on gross brain morphology after hypoxic–ischemic injury. Quantification of brain injury severity 72 h after HI injury. Dexamethasone pre-treatment significantly reduced the whitish discolored area in the ipsilateral hemisphere. Data are presented as mean ± SEM. One-way ANOVA with Tukey’s post hoc test. *** *p* < 0.001 vs. Vehicle group; ** *p* < 0.01 vs. Pre-treatment group. ns: not significant.

Macroscopic assessment of the brains 72 h after HI injury demonstrated that dexamethasone pre-treatment significantly reduced gross tissue damage. The mean area of whitish discoloration was significantly lower in the dexamethasone pre-treatment group (5.83 ± 14.29%) than in both the vehicle (55.50 ± 7.58%, *p* < 0.001) and post-treatment (47.08 ± 24.00%, *p* < 0.01) groups. In contrast, no significant difference in the discolored area was observed between the post-treatment and vehicle groups (*p* = 0.474). These findings suggest that dexamethasone pre-treatment effectively attenuates gross ischemic brain injury (Figure 1).

### 2.2. Pre-Treatment-Specific Transcriptomic Changes Associated with Dexamethasone-Mediated Neuroprotection

**Figure 2:**
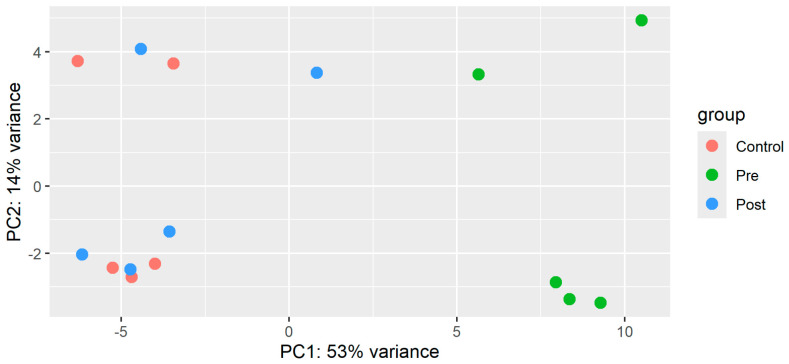
Principal Component Analysis (PCA) of RNA-seq data. PCA plot of global transcriptional profiles in the Vehicle, Post-treatment, and Pre-treatment groups (*n* = 5 per group). The Pre-treatment group formed a distinct cluster, whereas the Vehicle and Post-treatment groups largely overlapped.

To elucidate the molecular mechanisms underlying the neuroprotective effects of dexamethasone, transcriptomic profiling was performed in the vehicle, pre-treatment, and post-treatment groups. High-quality RNA samples were used for library preparation, and an average of 73.0 million reads per sample passed quality control and were used for subsequent analyses. Principal component analysis (PCA) revealed a clear separation of the pre-treatment group from the other two groups along the PC1 axis, which accounted for the largest variance in the dataset captured by the PC2 axis (Figure 2). Each point represents an individual biological replicate (*n* = 5 per group). In contrast, the vehicle and post-treatment groups exhibited closely clustered expression patterns, indicating that dexamethasone post-treatment exerted minimal impact on global gene expression and failed to induce a distinct transcriptomic shift compared with the hypoxic injury baseline. This distinct clustering suggests that dexamethasone pre-treatment induces a unique transcriptional reprogramming associated with neuroprotective mechanisms.

Using a nominal *p*-value threshold of <0.01, together with an absolute log2-fold change >2962 (407 upregulated and 555 downregulated) DEGs were identified between the pre-treatment and vehicle groups. In contrast, only 28 DEGs were detected between the post-treatment and vehicle groups.

**Figure 3:**
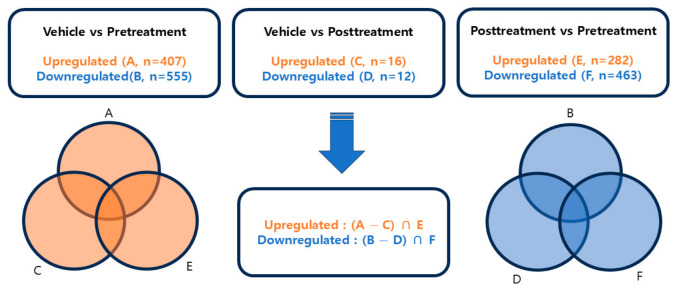
Schematic flow of the comparative transcriptomic analysis. Workflow illustrating the strategy for identifying key differentially expressed genes (DEGs) associated with dexamethasone-mediated neuroprotection. DEGs were first identified from three pairwise comparisons: vehicle vs. pre-treatment (A, B), vehicle vs. post-treatment (C, D), and post-treatment vs. pre-treatment (E, F). Candidate genes were then filtered using intersection analysis to isolate pre-treatment-specific effects. Genes with upregulated and downregulated expressions were defined as (A–C) ∩ E and (B–D) ∩ F, respectively, thereby excluding genes altered in the ineffective post-treatment condition. Orange indicates upregulated expression, whereas blue indicates downregulated expression.

As illustrated in Figure 3, pre-treatment-specific DEGs were identified using an intersection-based strategy.

First, we focused on genes that were uniquely regulated by pre-treatment. From the 407 genes with upregulated expressions in the pre-treatment group (Set A), we excluded 16 genes whose expressions were also upregulated in the post-treatment group (Set C). This yielded a subset of genes (A–C) specifically associated with the pre-treatment window. To further refine these candidates, we identified the intersection between this subset and the genes whose expressions were significantly upregulated in the pre-treatment vs. post-treatment comparison (Set E, 282 genes). This final intersection, (A–C) ∩ E, represents highly sensitive candidates whose expression is robustly increased specifically under neuroprotective conditions. Similarly, for DEGs with downregulated expressions, we identified the intersection between the pre-treatment-specific downregulated set (B–D) and the genes whose expressions were significantly downregulated in the pre-treatment vs. post-treatment comparison (Set F, 463 genes). Using this stringent filtering process, (B–D) ∩ F, allowed us to isolate a core set of genes that are potentially critical for dexamethasone-mediated neuroprotection while excluding non-specific transcriptomic changes observed in the ineffective post-treatment group. Furthermore, 745 DEGs were identified between the pre-treatment and post-treatment groups (282 upregulated and 463 downregulated, respectively). To identify key genes associated with neuroprotection, we focused on DEGs uniquely regulated in the pre-treatment group. By excluding genes also altered in the post-treatment group, a subset of pre-treatment-specific DEGs was identified, which may underlie the observed neuroprotective effects. The intersection analysis further highlighted genes highly responsive to dexamethasone pre-treatment.

### 2.3. Functional and Pathway Enrichment Analysis and Network Analysis

**Figure 4:**
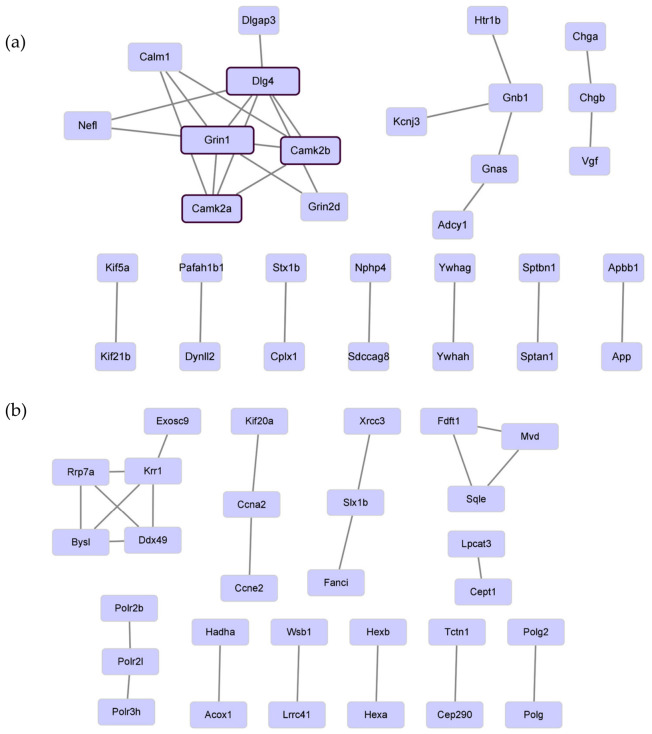
Protein–protein interaction (PPI) network analysis of DEGs. The network was constructed to provide a simplified visualization of key gene interactions based on known protein–protein associations. (**a**) Network analysis identified *Dlg4*, *Calm1*, and *Grin1* as highly connected hub genes within synaptic signaling pathways. (**b**) Downregulated genes did not show distinct clustering or central hub genes.

Functional and pathway enrichment analyses of DEGs were performed using the DAVID database based on Gene Ontology (GO) terms. Differential expression analysis between the control and pre-treatment groups identified 407 and 555 DEGs with upregulated and downregulated expressions, respectively. Functional enrichment analysis revealed 81 significantly enriched pathways (*p* < 0.05) among genes with upregulated expressions, of which 42 pathways remained significant at a more stringent threshold (*p* < 0.01). In contrast, DEGs with downregulated expressions were enriched in 19 pathways, predominantly associated with cellular metabolism, including lysosome organization, ribosome biogenesis, and lipid metabolic processes. Following intersection analysis (Figure 3), 195 ((A–C) ∩ E) and 384 ((B–D) ∩ F) genes with upregulated and downregulated expressions, respectively, were selected for further analysis. DEGs with upregulated expressions were predominantly enriched in pathways related to neuronal and synaptic functions. These pathways were broadly categorized into three major functional groups: nervous system development, ionic homeostasis, and synaptic signaling. Specifically, ionic homeostasis-related pathways included calcium ion transport, cellular calcium ion homeostasis, and membrane depolarization during action potentials. Synaptic function-related pathways encompassed regulation of synaptic plasticity, glutamatergic synaptic transmission, and dendritic morphogenesis. In addition, pathways involved in neuronal development, such as axonogenesis and neuron migration, were significantly enriched. Several core genes, including *Grin1*, *Dlg4*, and *Camk2a*, were repeatedly identified across multiple enriched pathways. In particular, *Grin1* showed the highest frequency of occurrence. Protein–protein interaction network analysis revealed a highly interconnected cluster among genes with upregulated expressions, consisting of 11 nodes. A central cluster involving *Dlg4*, *Calm1*, and *Grin1* exhibited high network connectivity (including betweenness centrality) (Figure 4a). The downregulated network formed smaller and less interconnected clusters, including a distinct module composed of *Ddx49*, *Krr1*, *Rrp7a*, and *Bysl* (Figure 4b).

### 2.4. qRT-PCR Analysis of Hub Genes

**Figure 5:**
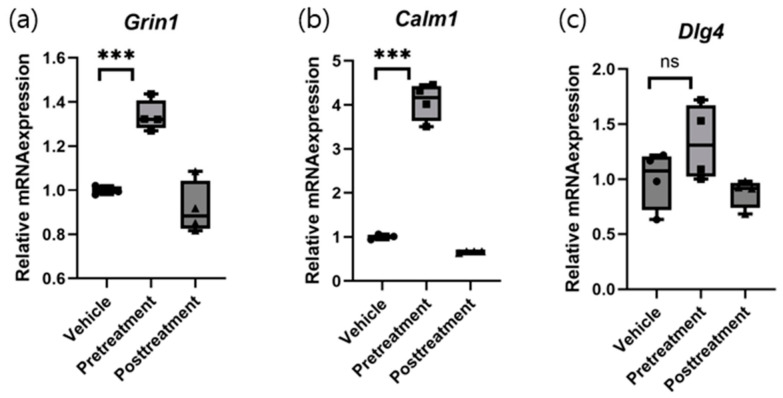
Validation of key hub genes by quantitative RT-PCR. Relative mRNA expression of *Grin1* (**a**), *Calm1* (**b**), and *Dlg4* (**c**) in the Vehicle, Pre-treatment, and Post-treatment groups (*n* = 4 per group). Data are presented as box-and-whisker plots. Statistical analysis was performed using one-way ANOVA with Tukey’s post hoc test. *** *p* < 0.001 vs. Vehicle group. ns: not significant.

qRT-PCR was used to validate the expression of key hub genes identified in the PPI network (*Dlg4*, *Grin1*, and *Calm1*; *n* = 4 per group). Consistent with the RNA-seq data, dexamethasone pre-treatment significantly increased the expression of *Grin1* (1.34 ± 0.04-fold; *p* < 0.001) and *Calm1* (4.08 ± 0.21-fold; *p* < 0.001) compared to the vehicle. Although *Dlg4* exhibited a similar upward trend in the pre-treatment group (1.33 ± 0.17-fold), the change did not reach statistical significance (*p* = 0.176) (Figure 5a–c). These findings are consistent with the gene expression patterns observed in the RNA-seq analysis. The concordance between transcriptomic and validation analyses supports the robustness of the findings despite the relatively small sample size.

## 3. Discussion

This study confirmed the neuroprotective effects of dexamethasone pre-treatment in a neonatal hypoxic–ischemic (HI) brain injury model. A key finding was the absence of efficacy in the post-treatment group, highlighting that the neuroprotective effect of dexamethasone is critically dependent on the timing of administration. This discrepancy underscores the importance of the therapeutic window in neonatal HI.

Given that HI injury triggers a rapid cascade of primary energy failure followed by secondary excitotoxicity, calcium dysregulation, and neuroinflammation, our findings suggest that dexamethasone may exert its protective effects via transcriptomic changes consistent with a preconditioning effect, stabilizing synaptic signaling pathways and maintaining calcium homeostasis prior to injury.

From a translational perspective, these findings suggest that dexamethasone may have greater potential as a preventive strategy by priming neural systems against impending injury, whereas its utility as a post-insult intervention appears limited. Transcriptomic and network analyses indicated that dexamethasone pre-treatment modulates gene expression in a pathway-specific manner, characterized by the upregulation of synaptic signaling pathways and calcium homeostasis and the downregulation of metabolic processes. The enriched pathways identified in the GO analysis were not isolated findings but were supported by network-level organization. Protein–protein interaction analysis showed that key genes, including *Dlg4*, *Grin1*, and *Calm1*, formed a tightly connected cluster, indicating coordinated regulation of synaptic and calcium signaling pathways. This convergence of pathway enrichment and network connectivity suggests that dexamethasone pre-treatment does not merely alter individual gene expression but reorganizes functionally coherent molecular networks involved in neuronal signaling. These findings further highlight a key mechanistic axis underlying dexamethasone-mediated neuroprotection, centered on calcium-dependent synaptic signaling, potentially involving NMDA receptor-associated pathways and downstream signaling molecules such as *Camk2a*. In contrast, genes with downregulated expressions did not form a distinct interaction network, further supporting the notion that dexamethasone selectively enhances organized synaptic pathways rather than broadly suppressing gene expression.

PPI network analysis identified several hub genes with upregulated expressions, including *Dlg4*, *Grin1*, and *Calm1*, which are pivotal in synaptic plasticity and calcium signaling [23,24]. Interestingly, upregulation of the expressions of NMDA receptor subunits (*Grin1*) and their scaffolding proteins (*Dlg4*) could be interpreted paradoxically. Although enhanced synaptic plasticity may support neuronal recovery, excessive NMDA receptor activation is a well-established driver of calcium-mediated excitotoxicity [25,26,27,28,29]. We hypothesize that dexamethasone-mediated upregulation during the pre-treatment phase may prime the neurons for survival or act as a mechanism of synaptic preconditioning, although the potential risk for late-stage excitotoxicity warrants further investigation. Conversely, the expressions of metabolism-related genes, including *Hexa*, *Hexb*, and *Idua*, were downregulated. This pattern resembles the metabolic suppression observed during therapeutic hypothermia, which is assumed to reduce cellular stress and energy demand [30,31]. However, as these genes encode essential lysosomal enzymes [32,33], the downregulation of their expression might reflect a benign reduction in metabolic demand. It could alternatively represent a harmful suppression of lysosomal degradation pathways, potentially leading to the accumulation of toxic byproducts like GM2 gangliosides [34]. Further proteomic studies are required to differentiate between protective metabolic conservation and pathological lysosomal inhibition.

In neonatal rats subjected to hypoxic–ischemic insults, a significant shift in the cytokine profile has been observed, characterized by an elevation of pro-inflammatory markers (IL-1β, IL-6, TNF-α) and a reduction in IL-10 [35]. In contrast, the neuroprotective effects observed in our study suggest that dexamethasone pre-treatment helps attenuate this cytokine imbalance. Such pre-insult modulation of the inflammatory milieu is likely to limit excessive inflammatory damage and promote a more permissive environment for neuronal and synaptic survival. The distinct effects observed between dexamethasone pre-treatment and post-treatment may reflect the temporal dynamics of hypoxic–ischemic injury. Early administration prior to HI insult may modulate early inflammatory and excitotoxic cascades, thereby preserving synaptic signaling and calcium homeostasis before secondary neuronal injury processes become fully established. In contrast, delayed administration after injury onset may have more limited effects, as downstream molecular and cellular damage pathways may already be activated. In this context, treatment administered after the onset of injury may fall outside the optimal therapeutic window, during which rapidly evolving inflammatory and oxidative stress responses may exceed the genomic regulatory capacity of corticosteroids. Despite the experimental neuroprotective evidence, dexamethasone is not currently recommended for clinical use in neonatal HI [36]. This is largely because of concerns regarding long-term neurodevelopmental side effects [37] and the narrow therapeutic window identified, which was also observed in this study. Previous studies have suggested that dexamethasone may exert timing-dependent effects in neural injury models. Early administration has been associated with reduced inflammatory and apoptotic responses, whereas delayed treatment has shown more limited effects. Although low-dose dexamethasone has generally demonstrated acceptable safety profiles in experimental settings, potential neurotoxic and developmental concerns have also been reported [38]. These findings support a more cautious interpretation of the present transcriptomic observations. The timing-dependent effects observed in this study further emphasize the need for careful consideration in clinical translation.

This study has some limitations. First, the conclusions are primarily based on transcriptomic and bioinformatic analyses; thus, direct confirmation of protein-level changes and functional activity remains to be established. However, the observed high degree of internal consistency across multiple pathway levels—particularly in synaptic signaling and calcium homeostasis—together with the identification of evolutionarily conserved hub genes, strongly supports the biological relevance of the inferred mechanisms. Although further functional validation using gene knockdown or pharmacological intervention is warranted, our findings provide a robust molecular framework that may help narrow down potential therapeutic targets for neonatal HI brain injury. Second, because of the exploratory nature of this study and limited sample size, statistical power may have been reduced, and the possibility of false-positive findings cannot be excluded.

Third, direct histological assessments, such as 2,3,5-triphenyltetrazolium chloride (TTC) staining or Nissl staining, as well as brain-to-body weight measurements, were not systematically performed in the current study. Therefore, the present findings should be interpreted primarily as exploratory molecular evidence rather than definitive structural confirmation of tissue preservation. Additional studies incorporating histological and long-term functional evaluations are warranted to further validate the neuroprotective effects of dexamethasone pre-treatment. Fourth, sex-specific differences were not systematically analyzed, despite the well-documented variability in HI susceptibility between males and females. Fifth, this study focused on the acute phase (72 h) following HI injury; therefore, further investigations are needed to assess the long-term effects of dexamethasone on neurodevelopmental and behavioral outcomes. Sixth, the absence of a sham-operated group means that a true baseline transcriptomic profile of the healthy neonatal brain was not established. Although the vehicle-treated HI group served as an appropriate injury control, future studies including sham controls are necessary to account for potential surgical or anesthetic effects. Nevertheless, using the vehicle-treated HI group as a reference, we focused on identifying the transcriptomic shifts specifically associated with the timing of dexamethasone administration. Finally, the assessment of neuroprotection relied primarily on gross morphological evaluation (whitish discoloration) and transcriptomic profiling. Although blinded assessments were performed, this approach lacks the resolution of gold-standard histological methods. Moreover, although the neonatal HI mouse model is widely used, caution is warranted when extrapolating these findings to clinical settings because of interspecies physiological differences.

In summary, although these limitations should be considered when interpreting our findings, the present study provides a preliminary molecular framework for understanding the time-dependent effects of dexamethasone in the neonatal brain. Future studies incorporating histological validation (e.g., TTC or Nissl staining), as well as protein-level validation, are required to confirm the structural and functional relevance of our transcriptomic observations.

## 4. Materials and Methods

### 4.1. Establishment of an Animal Model of Neonatal HI Brain Injury

All experimental procedures were approved by the Institutional Animal Care and Use Committee of Yonsei University College of Medicine, Seoul, Korea (Permit No. 2015-0378; approved on 2 February 2016) and were conducted in accordance with the ARRIVE 2.0 guidelines [39].

The Rice–Vannucci model of neonatal hypoxic–ischemic brain injury was employed as previously described [40]. Postnatal day 7 (P7) Institute of Cancer Research mice were subjected to permanent occlusion of the right common carotid artery under isoflurane anesthesia. Anesthesia was induced with 3–4% isoflurane and maintained at 1–2% in oxygen during the surgical procedure. The duration of anesthesia was limited to approximately 5–10 min for carotid artery ligation to minimize potential anesthetic effects on neuronal injury and gene expression. Following surgery, unilateral HI brain injury was induced by exposure to 8% oxygen balanced with nitrogen for 70 min at 37 °C on a heating plate within a closed chamber. Animals were housed under a 12 h light/dark cycle in accordance with institutional animal care regulations. Animals were euthanized on postnatal day 10 by an overdose of isoflurane (5% in oxygen) followed by decapitation in accordance with institutional and ethical guidelines. No animals were excluded because of mortality or experimental failure.

### 4.2. Treatment with Dexamethasone Administration

The pups were randomly assigned to three experimental groups. (1) Group A: vehicle (*n* = 5); (2) Group B: dexamethasone pre-treatment (*n* = 6); (3) Group C: dexamethasone post-treatment (*n* = 6). In dexamethasone-pretreated animals, a 0.5 mg/kg dose of dexamethasone was administered via intraperitoneal injections 6 h before HI brain injury, and an equal volume of phosphate-buffered saline (PBS) was administered 6 h after HI brain injury. In dexamethasone post-treated animals, PBS was administered via an intraperitoneal injection 6 h before HI brain injury and a 0.5 mg/kg dose of dexamethasone was administered 6 h after HI brain injury. In the vehicle group, the same volume of PBS was injected intraperitoneally 6 h before and after HI brain injury.

Using the vehicle-treated HI group without a sham group as a reference, we focused on identifying the transcriptomic shifts specifically associated with the timing of dexamethasone administration.

### 4.3. Effects of Dexamethasone on Gross Brain Morphology After Hypoxic–Ischemic Injury

To evaluate the potential neuroprotective effects of dexamethasone pre-treatment, HI brain injury was assessed by gross inspection. Mice were euthanized 72 h after HI injury, deeply anesthetized, and transcardially perfused with cold PBS. The skull was opened along the midline, and the brain was carefully removed from the cranial cavity to avoid mechanical damage. Gross morphology was immediately examined. The severity of the injury was macroscopically evaluated by determining the percentage of the grossly visible whitish area relative to the total area of the ipsilateral hemisphere. All assessments were performed independently by two investigators blinded to the experimental groups. The estimated percentage was used for group comparisons.

### 4.4. RNA Extraction and RNA-Seq Library Construction

For transcriptomic analysis, RNA sequencing was performed on a subset of samples (*n* = 5 per group) selected based on RNA quality and sample consistency. The ipsilateral hemispheres were dissected and homogenized using Precellys Lysing kits (Bertin Technologies, Montigny-le-Bretonneux, France; Cat. #CK14) and a Precellys 24 tissue homogenizer (Bertin Technologies, Montigny-le-Bretonneux, France) in Tri Reagent (Molecular Research Center, Cincinnati, OH, USA) for total RNA extraction. RNA quality and purity were verified via spectrophotometry; all samples exhibited A260/280 and A260/230 ratios of approximately 2.0, indicating high purity. Although RNA integrity number values were not formally recorded, sample integrity was ensured because of consistent optical density ratios and careful handling during extraction. Five samples per group were selected for RNA sequencing. RNA libraries were prepared using the TruSeq Stranded mRNA Library Prep Kit (Illumina, San Diego, CA, USA), and sequencing was performed by Macrogen Co. (Seoul, Republic of Korea) on an Illumina HiSeq 2000 platform, according to the manufacturer’s instructions. Raw sequencing data were processed using the Illumina pipeline (Phred+33 encoding). A total of 15 samples generated an average of 74.3 million paired-end reads (101 bp) per sample. Following quality filtering, an average of 73.0 million clean reads per sample was retained, with a mean GC content of 52.1% and Q30 scores exceeding 95.8%.

### 4.5. Data Preprocessing and DEG Identification

All primary analyses of RNA-seq data were performed using STAR (version 2.7) and DESeq2 (version 1.26). RNA-seq reads were aligned to the mm10 reference genome using STAR. Gene-level read counts were generated using HTSeq based on the annotated gene models. Differential expression analysis was conducted using DESeq2, which employs a negative binomial distribution model. Pairwise comparisons were performed between the vehicle and each treatment group to identify DEGs. FDR-adjusted *p*-values were initially calculated using the Benjamini–Hochberg method to account for multiple testing. However, application of a stringent FDR-adjusted significance threshold yielded only a limited number of detectable DEGs, likely reflecting reduced statistical power associated with the relatively small sample size and biological variability inherent to the neonatal hypoxic–ischemic injury model. Therefore, for exploratory transcriptomic profiling aimed at identifying candidate molecular pathways associated with timing-dependent dexamethasone effects, downstream DEG selection was performed using a nominal *p*-value threshold (*p* < 0.01) together with a stringent fold-change criterion (|log2FC| > 2).

This alternative filtering strategy was adopted to preserve sensitivity for detecting coordinated transcriptional alterations while minimizing biologically trivial findings. Importantly, the transcriptomic findings were interpreted in conjunction with downstream pathway enrichment analysis, protein–protein interaction network analysis, and qRT-PCR validation of representative hub genes to further support their biological relevance. In addition, transcriptomic patterns were cross-referenced with a publicly available Gene Expression Omnibus (GEO) dataset (Accession No. GSE325742). Given the exploratory nature of the present study, the identified transcriptomic alterations should be interpreted as hypothesis-generating molecular signatures rather than definitive mechanistic evidence. The list of DEG tables based on different FDR thresholds has been updated as Supplementary Material. Detailed gene lists are provided in Supplementary File S1.

### 4.6. Pathway Enrichment and Protein–Protein Interaction (PPI) Network Analyses

Pathway enrichment and PPI network analyses were performed using the exploratory DEG set identified from the nominal *p*-value threshold. GO enrichment analysis was conducted using the DAVID database (https://davidbioinformatics.nih.gov/, accessed on 26 May 2026) based on a hypergeometric distribution model (*p* < 0.01). PPI networks were constructed using the STRING database (version 9.1; https://string-db.org/, accessed on 26 May 2026), which integrates experimentally validated and predicted protein interactions derived from multiple sources, including experimental evidence, co-expression, and curated databases. A high-confidence interaction score threshold (confidence score >0.9) was applied to minimize low-confidence interactions. The resulting PPI networks were visualized using Cytoscape software (version 3.10.4; https://cytoscape.org/, accessed on 26 May 2026). Proteins were represented as nodes, and interactions between proteins were represented as edges. Node degree was defined as the number of interactions per protein, and hub proteins were identified based on relatively high node degrees.

### 4.7. Validation of Expression Changes in Key Genes Using qRT-PCR

qRT-PCR conducted using a further subset (*n* = 4 per group) was used to validate the expression levels of selected genes with upregulated expressions (*Dlg4*, *Grin1*, and *Calm1*). RNA concentration was measured using a NanoDrop spectrophotometer, and 1 μg of total RNA was reverse-transcribed into cDNA using a first-strand cDNA synthesis kit (Roche, Basel, Switzerland) according to the manufacturer’s instructions. The synthesized cDNA was stored at −20 °C until use. qRT-PCR was performed in 384-well plates using a LightCycler 480 System (Roche). Each 10 μL reaction contained 0.5 μL of cDNA and LightCycler 480 SYBR Green I Master Mix (Roche). The amplification conditions were as follows: initial denaturation at 95 °C for 5 min, followed by 45 cycles of 95 °C for 10 s, 60 °C for 20 s, and 72 °C for 15 s. A melting curve analysis was performed to confirm primer specificity. Relative gene expression levels were normalized to Gapdh. Gapdh was used as the internal reference gene, as its expression remained stable across all experimental groups in the RNA-seq dataset (Table 1). Gene expression was calculated using the 2^−ΔΔCt^ method [41]. All primer sequences and expected product sizes are listed in Table 1.

### 4.8. Statistical Analysis

All statistical analyses were performed using SPSS software (version 25; IBM Corp., Armonk, NY, USA). Comparisons between two groups were conducted using the Mann–Whitney U test, whereas comparisons among multiple groups were performed using one-way analysis of variance (ANOVA), followed by Bonferroni post hoc tests for pairwise comparisons. A formal power analysis was not conducted; instead, the sample size was determined based on previous similar studies and ethical considerations to minimize animal use. Data are presented as mean ± standard error of the mean (SEM), and *p* < 0.05 was considered statistically significant.

## 5. Conclusions

Dexamethasone pre-treatment was associated with time-dependent neuroprotection in neonatal hypoxic–ischemic brain injury by preserving synaptic and calcium-dependent signaling networks while suppressing metabolic pathways. These transcriptomic changes suggest that maintenance of synaptic integrity and calcium homeostasis may be associated with potential neuroprotective mechanisms following hypoxic–ischemic injury. Our findings underscore the potential importance of early intervention and support the existence of a narrow therapeutic window for dexamethasone administration. Although this study provides novel molecular insights, further functional studies are required to translate these transcriptomic findings into clinical applications.

## Figures and Tables

**Table 1:** Primer sequences and conditions for quantitative real-time polymerase chain reaction analysis.

Primer Name	Primer Sequence (5′-3′)	Primer Name	Primer Sequence (5′-3′)
Gapdh_F	AGATGGTGATGGGCTTCCC	Gapdh_R	GGCAAATTCAACGGCACAGT
*Dlg4*_F	CAACGACAGCATCCTGTTGTC	*Dlg4*_R	TCCACTGCAGCTGAATGGGT
*Calm1*_F	ACAGATAGCGAAGAAGAGATCCGC	*Calm1*_R	TCTGCCGCACTGATGTAACCATTCC
*Grin1*_F	TCTTCATGCTGGTGGCTGGA	*Grin1*_R	TTGTGTCGCTTGTAGGCGAT

Specific primers were designed to validate the expression of hub genes identified from the PPI network analysis. Gapdh was used as an internal housekeeping gene for normalization. Forward and reverse primer sequences are shown in the 5′ to 3′ direction. All reactions were performed in triplicate to ensure reproducibility. Gapdh, glyceraldehyde-3-phosphate dehydrogenase; Dlg4, disks large membrane-associated guanylate kinase scaffold protein 4; Grin1, glutamate ionotropic receptor NMDA type subunit 1; Calm1, calmodulin 1.

## Data Availability

The data discussed in this publication have been deposited in NCBI’s Gene Expression Omnibus [42] and are accessible through GEO Series accession number GSE325742 (https://www.ncbi.nlm.nih.gov/geo/query/acc.cgi?acc=GSE325742, accessed on 26 May 2026).

## Supplementary Materials

The following supporting information can be downloaded at: https://www.mdpi.com/article/10.3390/ijms27114920/s1.

## Abbreviations

The following abbreviations are used in this manuscript:

HI	Hypoxic–ischemic
PI3K/Akt	Phosphatidylinositol-3-kinase/Akt
NGS	Next-generation sequencing
RNA-seq	RNA sequencing
DEGs	Differentially expressed genes
RT-qPCR	Reverse transcription quantitative polymerase chain reaction
OCT	Optimal cutting temperature
PCA	Principal component analysis
ANOVA	Analysis of variance
SEM	Standard error of the mean
CaMK2	Calcium/calmodulin-dependent protein kinase II
TTC	2,3,5-triphenyltetrazolium chloride
